# The new nordic diet – consumer expenditures and economic incentives estimated from a controlled intervention

**DOI:** 10.1186/1471-2458-13-1114

**Published:** 2013-12-02

**Authors:** Jørgen Dejgård Jensen, Sanne Kellebjerg Poulsen

**Affiliations:** 1 Department of Food and Resource Economics, University of Copenhagen, Rolighedsvej 25, DK-1958, Frederiksberg C, Denmark; 2 Deparment of Nutrition, Exercise and Sports, University of Copenhagen, Frederiksberg C, Denmark

**Keywords:** Consumer expenditure, Market incentives, New nordic diet

## Abstract

**Background:**

Several studies suggest that a healthy diet with high emphasis on nutritious, low-energy components such as fruits, vegetables, and seafood tends to be more costly for consumers. Derived from the ideas from the New Nordic Cuisine – and inspired by the Mediterranean diet, the New Nordic Diet (NND) has been developed as a palatable, healthy and sustainable diet based on products from the Nordic region. The objective of the study is to investigate economic consequences for the consumers of the NND, compared with an Average Danish Diet (ADD).

**Methods:**

Combine quantity data from a randomized controlled ad libitum dietary 6 month intervention for central obese adults (18–65 years) and market retail price data of the products consumed in the intervention. Adjust consumed quantities to market price incentives using econometrically estimated price elasticities.

**Results:**

Average daily food expenditure of the ADD as represented in the unadjusted intervention (ADD-i) amounted to 36.02 DKK for the participants. The daily food expenditure in the unadjusted New Nordic Diet (NND-i) costs 44.80 DKK per day per head, and is hence about 25% more expensive than the Average Danish Diet (or about 17% when adjusting for energy content of the diet). Adjusting for price incentives in a real market setting, the estimated cost of the Average Danish Diet is reduced by 2.50 DKK (ADD-m), compared to the unadjusted ADD-i diet, whereas the adjusted cost of the New Nordic Diet (NND-m) is reduced by about 3.50 DKK, compared to the unadjusted NND-i. The distribution of food cost is however much more heterogeneous among consumers within the NND than within the ADD.

**Conclusion:**

On average, the New Nordic Diet is 24–25 per cent more expensive than an Average Danish Diet at the current market prices in Denmark (and 16–17 per cent, when adjusting for energy content). The relatively large heterogeneity in food costs in the NND suggests that it is possible to compose an NND where the cost exceeds that of ADD by less than the 24–25 per cent.

## Background

Several studies and reviews [1-3] suggest that a healthy diet with high emphasis on nutritious, low-energy components such as fruits, vegetables, and seafood tends to be more costly for consumers, due to the general tendency for low-cost foods to be low in nutrients but high in energy. For this reason, especially low-income consumers tend to compose their diet of foods with poor nutritional quality to a larger extent than higher-income consumers, and this may constitute an important barrier for consumers’ switch towards such healthier diets, especially in economically and socially deprived households [3].

Detailed studies have addressed this issue in relation to nutrition for the population at large in different countries, based on dietary survey data or cross-section questionnaire data, by estimating the costs of a diet adhering to a high Healthy Eating Index or similar measures [4-6] or to a specific regional diet, e.g. a Mediterranean diet [6-8]. The studies tend to find that such diets are more costly than diets with higher energy density and lower nutritional quality in the respective populations.

Since the turn of the century, the concept of New Nordic Cuisine has been developed, as formulated in a Manifesto from 2003 [9], and the concept has been relatively successful in gaining ground in Nordic gourmet restaurants [10,11]. The concept emphasizes environmental sustainability resulting from presumed less transportation of food products, use of seasonal produce and exploitation of food resources from the wild countryside.

Derived from the ideas from the New Nordic Cuisine – and inspired by the Mediterranean diet [12], the New Nordic Diet (NND) has been developed as a palatable, healthy and sustainable diet based on products from the Nordic region. NND was developed within the Danish OPUS project, and the principles of the New Nordic Diet have been delineated in Mithril et al. [13]. Overall, the NND is described by the overall guidelines: (i) more calories from plant foods and fewer from meat; (ii) more foods from the sea and lakes; and (iii) more foods from the wild countryside.

Against this background, the objective of the present study is to investigate the economic consequences for the consumers of the New Nordic Diet, compared with the Average Danish Diet (ADD). In particular, it is investigated, to which extent the New Nordic Diet is more costly than the Average Danish Diet, and to identify some of the key elements in such cost differences. The study is based on data from a 6 months intervention study conducted under the auspices of the OPUS project.

## Methods

With the objective of estimating the costs associated with alternative dietary patterns, two main approaches have been used in the literature. In one approach, observational data on dietary patterns, from e.g. dietary surveys [4-6,8,14-17] or questionnaire surveys [18,19] have been used to determine nutritional characteristics, and in combination with retail price data to calculate the dietary costs, at individual – or household – level. Based on such data and calculations, it is possible to investigate correlations between costs and nutritional characteristics, e.g. adherence to a specified dietary quality. The other approach takes departure in dietary interventions, where participants are assigned to e.g. an intervention diet or a control diet, and the costs of each diet is calculated by combining data on food quantities with retail price data [7,20-22]. In the present study, we investigate the consumer expenditure on the basis of quantity data from a 6 months dietary intervention [23], combined with market retail price data of the products consumed in the intervention.

### The 6 months dietary intervention

The New Nordic Diet holds ambitions in three dimensions. First, it should contribute to the *prevention of health disorders* such as weight gain, type 2 diabetes, cardiovascular diseases and cancer, but should also help maintaining and improving general physical, mental and social well-being through a lower meat intake and a higher intake of legumes, vegetables, fruit, whole grains, seafood, potatoes, nuts, herbs, etc. than the average Danish diet. Second, it should *utilize and develop the gastronomic potential* and Nordic identity, based on food with a Nordic origin and cultural heritage. Tastes from arctic fish, shellfish and seaweed, and colour and flavor variation from plant foods, such as berries, cabbages, roots, legumes, potatoes and herbs contribute to creating a Nordic identity of the dishes. Third, the diet should be *sustainable by use of locally grown foods* to minimize transport of food stuffs, use of organic products, use of foods sourced from the wild countryside, shift in consumption from meat to plant products and focus on minimizing food waste contribute to reducing environmental strains from food production.

Compared with the average Danish diet, the New Nordic Diet represents some fundamental changes, mainly in terms of a substantially higher emphasis on vegetables, whole grains, seafood and wild ingredients, and lower emphasis on meat (see Table 1).

**Table 1:** Overview of the average daily content of the dietary components in the New Nordic Diet (NND) in relation to the average daily content in the Danish population (energy-adjusted intake (per 10 MJ) of all persons aged 4–75 years

**Dietary component**	**Average content in the NND (g/day)**	**Content in the ADD (g/day)**	**Average content in the Danish population (g/day)**
**Ingredients, g/10 MJ**			
Fruit	>300 *(250–350)*	*150-250*	240
Vegetables	>400 *(350–450)*	*150-210*	181
Including			
- berries	*(50–100)*	*2-6*	5
- cabbages	>29 *(25–35)*	*<=10*	9
- root vegetables	*>150*	*25-35*	38
- legumes	*>30*	*<=1*	7
Fresh herbs	As much as possible *(> = 1)*	*< 1*	
Potatoes	>140 *(140–160)*	*90-110*	106
Plants and mushrooms from the wild countryside	5 *(3–7)*	*0*	<1
Whole grains	*>75*	*25-45*	36
Nuts	*>30*	*<=1*	1
Fish and shellfish	>43 *(40–50)*	*15-25*	22
Seaweed	5 *(3–7)*	*0*	<1
Free-range livestock	85-100 *(90–110)*	*130-150*	143
Including			
- game	>4 *(2–6)*	*0*	<1
**Macronutrients etc.**			
Protein (E%)	18 *(15–23)*	*10-20*	15
Total carbohydrate (incl. fibres), E%	52 *(48–56)*	*45-50*	50
Added sugar (E%)	*<10*	*> = 12*	
Total fat (E%)	30 *(25–35)*	*33-37*	35
Saturated fat (E%)	*<10*	*10-20*	15
Nordic produce (%)	*> = 95*	*<=50*	
Organic (%)	*> = 50*	*<=10*	

Italics: Limits imposed in the intervention.

E%: Energy per cent.

Source: [24].

The intervention was a randomized controlled ad libitum dietary intervention for central obese adults (18–65 years), and a majority of the participants with one or more components of the metabolic syndrome [25]. After a screening, eligible participants were stratified upon BMI, age and whether the participant was part of a couple, where the spouse also participated.

In the study, 181 centrally obese adults were recruited for a 28 weeks dietary intervention and randomized to one of two diets using simple bloc randomization. 147 participants completed the intervention. In the present cost analysis, we focus on participants who did not have a spouse participating, in order to minimize potential problems in the assessment of consumed food quantities. 99 of the completing participants fulfilled this requirement, and of these, 40 followed an Average Danish Diet (ADD) reflecting the average dietary composition of food consumption [26], and 59 followed a New Nordic Diet (NND), building on the principles outlined above.

In either diet, participants were provided with food commodities for free from a study shop at the Department of Human Nutrition at the University of Copenhagen. In the shop, participants had the self-selected commodities registered in a web-based computer application designed for the study in order to check that the composition of foods was consistent with the prescribed diet (e.g. in terms of energy composition, geographic origin of commodities, organic or not, etc.), and if not, they were told to adjust their choices. All “purchases” were recorded on each “shopping session”, and these recordings constitute the base data regarding consumption quantities. In order to assist the participants in following the respective diets, they were provided with diet-specific sets of recipes. Along with participating in the shop experiment, participants were also required to participate in physical examinations before, during and after the intervention, and to regularly receive dietary advice from a dietician. The first participants started in September 2010, and the last participants completed in July 2011. Permission to use the data from the experiment for the present study was granted by the collectors of data, Sanne Kellebjerg Poulsen and Thomas Meinert Larsen, Department of Sports and Human Nutrition, University of Copenhagen.

It was expected that the participants’ choice of food products in the intervention could be flawed by the fact that they got all their food products for free in the shop. Compared to a ‘real’ market situation, where consumers have to pay the price of the commodities, this intervention design may have implied an incentive to consume more of (normally) high-priced commodities and less of low-price commodities than if the participants were paying the market price of the commodities, and hence that the observed choices may represent a biased picture of the likely behavior in a normal market setting. In order to correct for this, we have established a modified version of the ADD and NND, where such price incentives have been taken into account. Consequently, we analyze the consumer expenditures of four alternative diets: Average Danish Diet (ADD-i), New Nordic Diet (intervention) (NND-i), as well as estimates of these two diets under market conditions (ADD-m and NND-m).

### Quantity data from the intervention

The implementation of the NND was formulated to largely follow the Nordic Nutrition Recommendations [27] with regard to the composition of macronutrients, whereas the macronutrient composition of the ADD was formulated to be similar to that found in recent dietary surveys [26]. In addition to macronutrient composition, the two diets were also distinguished by their composition of ingredients, cf. Table 1.

Ingredients in the NND primarily consisted of Danish/Nordic produced commodities and commodities that were in season. Furthermore, it was an aim that 75 per cent of the intake should be organically produced. The dietary intake was expected to be ad libitum, and the guidance of the participants was primarily focused on the composition of the diet, rather than the amount of energy. Proposed season-specific menu plans with recipes were handed out to the participants, and they were recommended to follow this menu plan as closely as possible. In the ADD, ingredients were a mixture of domestic and imported products, the seasonal variation was lower and organic products were not included. Also for the ADD, an ad libitum intake was expected and guidance was primarily related to diet composition rather than total energy intake. Participants were also offered recipes, but with no specific menu plans.

### Market price data

The majority of the market prices facing the consumers (representing more than 90 per cent of the total dietary expenditure in both diet scenarios) have been estimated on the basis of household purchase data from the GfK Scandinavian Consumer tracking panel, which is a demographically representative consumer panel from all the different regions of Denmark. The data used covers 2010 and is an unbalanced panel that contains approximately 3000 households. Panel households keep detailed diaries of shopping on a weekly basis. For each shopping trip, the diary-keeper reports purchases of foods and other staples including the date and time of the purchase, the name of the store and the total expenditure on the shopping trip. For almost all goods in all periods, the value and quantity of the product is recorded. Permission to use the GfK data for the present study is granted by a general contract between the GfK company the Department of Food and Resource Economics, University of Copenhagen. Average unit prices for individual months have been calculated as the ratio between average value and average quantity of these purchases.

As not all ingredients of the two diets are monitored in the GfK data material, supplementary data have been collected from a variety of sources, including web shops and physical food stores. Some of the ingredients could only be found in very few (in some cases only one) shops, and hence the robustness of these price estimates is considerably lower than is the case for most of the prices estimated on the basis of GfK data. However, such more “rare” food ingredients constitute a relatively limited share of the budgetary cost in the two diets, and the influence of the uncertainty regarding these prices on the total dietary cost is relatively limited.

A few “wild” plants (e.g. dandelions, nettles, goutweed), are not currently on the commercial market to an extent that enables estimation of market prices. For such plants, it is assumed that a market price in a commercial production would be similar to that of green cabbage.

### Estimation of NND-m and ADD-m diets on market conditions

As the participants got their foods for free, no direct price incentive was reflected in their food choices. However, their choices were made subject to certain restrictions as defined by the respective diets, including restrictions on the dietary composition, commodities of Nordic origin, share of commodities organically produced etc. (cf. Table 1). Hence, although no direct incentives were in place, these restrictions provided some implicit “price incentives”, reflecting the bindingness of the respective restrictions. For example, a minimum requirement to the total intake of cabbage would imply that a one gram decrease in the intake of one type of cabbage must be compensated by a corresponding increase in the intake of another type of cabbage, etc. This would imply an equal implicit price per gram of all cabbages. The observed data can thus be interpreted as the solution to the participants’ traditional utility maximization problem, if the budget line was determined by these implicit prices.

If instead the participants should buy their foods at existing market prices, $p_{j}^{*}$, they would have incentives to compose their foods differently, buying more products with a low price per gram and less products with a high price per gram. Given the observed consumption from the intervention, $x_{j}^{D-i}$, and the price elasticity of demand, *ε*_*j*_, an estimate of the price-adjusted consumption, $x_{j}^{D-m}$, in diet *D* can be determined as

$$x_{j}^{D-m}\;\approx \;x_{j}^{D-i}\cdot {\left(p_{j}^{*}/{\hat{p}}_{j}\right)}^{\epsilon_{j}}$$

Provided these estimated quantities in a market setting, consumer expenditures can be estimated in the respective scenarios: $E^{D-i}=\sum p_{j}^{*}\cdot x_{j}^{D-i}$ and $E^{D-m}=\sum p_{j}^{*}\cdot x_{j}^{D-m}$, *D* ∈ {*NND*, *ADD*}.

Price elasticities for the calculation of “market” quantities were estimated econometrically on the basis of the above-mentioned GfK data describing households’ purchases. In particular, the following linear regression equation was formulated, specifying the household *f*’s demanded quantity of commodity *i* in time period *t* ($x_{\mathrm{it}}^{f}$) as a log-linear function of the logarithmic price of this commodity. The model is estimated as a fixed-effect model, implying that we regress quantity deviations from household means on price deviations from household means.

$$\ln x_{\mathrm{it}}^{f}-\bar{\ln x_{\mathrm{it}}^{f}}=\alpha_{i}+\epsilon_{\mathrm{ii}}\cdot \left(\ln p_{\mathrm{it}}^{f}-\bar{\ln p_{\mathrm{it}}^{f}}\right)+u_{\mathrm{it}}^{f}$$

“Raw” household-level price data contained a lot of missing values (because not all commodity types are bought every month by every household), which is a problem for the estimation, as the price information is also important in “no-purchase” months. For this reason, we replaced raw prices with “synthetic” household-level commodity prices defined as ${\hat{p}}_{\mathrm{jt}}^{f}=\varphi_{j}^{f}+\varphi_{\mathrm{jp}}\cdot p_{\mathrm{jt}}$, where *P*_*jt*_ is an average of the price variable across households in month *t* ($=\sum_{h}p_{\mathrm{jt}}^{f}\cdot x_{\mathrm{jt}}^{f}/\sum_{h}x_{\mathrm{jt}}^{f}$), and $\varphi_{j}^{f}$ and *ϕ*_*jp*_ are parameter estimates from a fixed-effect linear regression of observed household-level prices on the constructed average price variable.

An illustration of the dietary adjustment to price incentives using the estimated price elasticities for the group of root vegetables is given in Table 2.

**Table 2:** Prices, price elasticities and average correction to market prices for root vegetables

	**Price (DKK/kg)**	**Price elasticity**	**Quantity adjustment**
Carrot	6.36	−0.46	47%
Potato	6.81	−0.76	79%
Onion	7.62	−0.23	16%
Parsnip	23.11	−0.41	−17%
Radish	43.24	−0.41	−36%
Beet root	21.03	−0.23	−8%
Celery root	16.97	0.00	0%
Fennel	72.37	−0.26	−34%
Fennel, organic	98.08	−0.74	−68%
Carrot, organic	9.25	−0.22	19%
Shallot, organic	13.63	−0.39	17%
Beet root, organic	34.77	−0.53	−24%
Hamburg parsley, organic	45.84	−0.88	−5%
Hamburg parsley	33.73	−0.80	−33%
Radish, organic	64.22	−0.88	−63%
Frozen root mix	23.58	−0.19	−3%
Parsnip, organic	30.05	−0.79	−26%
Average root vegetable price, ADD	14.66		
Average root vegetable price, NND	20.53		

Note. Price elasticity: Per cent change in consumption due to 1 per cent increase in price.

For example, the average price of root vegetables in the ADD is 14.66 DKK/kg, whereas the market price of non-organic carrots is 6.36 DKK/kg, implying that if the consumer is faced with the market prices, she will have an incentive to use more carrots within the group of root vegetables than if she would be paying the same price for all root vegetables. With the estimated price elasticity of −0.46, this implies a 47% upward adjustment in the consumption of carrots. It should be noted that we ignore potential cross-price substitution effects in this adjustment, for example that the adjustment to the lower price for carrots might affect the consumption of potatoes and onions differently. Although this simplification may impose a bias on the estimated adjustment, we consider this bias to be minor.

## Results

Market prices were estimated on a monthly basis using the procedure outlined above. As expected, som of the prices, for example fresh fruits and vegetables, exhibited significant seasonal variation, whereas many others had relatively stable prices through the year.

Combining the estimated prices with the reported quantities from the intervention, it is possible to calculate the daily food expenditure, if the participants would have to buy the food commodities in the normal retail market. These calculated costs are displayed in Table 3, based on the consumed quantities reported in the intervention (ADD-i, NND-i) as well as the behaviourally adjusted quantities if consumers were facing the real market prices (ADD-m, NND-m).

**Table 3:** Average daily food expenditure

**DKK/head/day**	**ADD-i**	**ADD-m**	**NND-i**	**NND-m**
Milk and cheese	5.58	4.55	4.00	3.50
Grain products	4.73	4.21	5.73	5.53
Vegetables	4.67	4.24	13.07	10.76
Fruits and berries	4.78	4.69	5.94	5.90
Meat	6.89	6.51	3.07	2.57
Seafood	1.53	1.56	6.22	6.16
Poultry meat and eggs	1.49	1.46	1.20	1.14
Butter. oils. etc.	0.69	0.67	0.48	0.42
Sugar	2.80	2.77	0.86	0.84
Non-alcoholic beverages	0.83	1.04	2.31	2.23
Alcholic beverages	0.64	0.64	1.11	1.04
Spices. seasonings etc.	1.40	1.15	0.83	1.15
Total	36.02	33.50	44.80	41.24
Total per 10 MJ energy intake	46.00	45.06	54.03	52.11

Note

ADD-i: Average Danish Diet with consumed quantities as measured in intervention.

ADD-m: Average Danish Diet with consumed quantities adjusted for market price differences.

NND-i: New Nordic Diet with consumed quantities as measured in intervention.

NND-m: New Nordic Diet with consumed quantities adjusted for market price differences.

According to Table 3, the average daily food expenditure of the Average Danish Diet as represented in the unadjusted intervention (ADD-i) amounted to 36.02 DKK for the participants in the intervention (if they were paying). Dairy products, grain products, meat, fruits and vegetables constitute significant shares of this daily expenditure, with about 15–20 per cent of the expenditure each. Sugar and sugar products also represent a significant share of the ADD, by 7–8 percent of the budget. The NND’s higher emphasis on vegetables and seafood and lower emphasis on meat and sugar (cf. Table 1) is also clearly reflected in the cost figures, where the former elements constitute a larger share of the budget than in the ADD, whereas meat and sugar represent a lower daily expenditure than in the ADD. The daily food expenditure in the unadjusted New Nordic Diet (NND-i) costs almost 45 DKK per day per head, and is hence about 9 DKK (or about 25%) more expensive than the Average Danish Diet.

Taking into account the price incentives in a real market setting, where consumers would tend to opt for lower-priced commodities instead of high-price goods to some extent (within the above-mentioned guidelines for the respective diets), the estimated food expenditure becomes lower in both diets. In particular, the estimated cost of the Average Danish Diet is reduced by 2.50 DKK (ADD-m), compared to the unadjusted ADD-i diet, whereas the adjusted cost of the New Nordic Diet (NND-m) is reduced by about 3.50 DKK, compared to the unadjusted NND-i, with the main economic savings found in the group of vegetables, but also some in dairy and meat products. In the bottom of Table 3, results for an energy-adjusted diet (10 MJ/day) are presented, showing that the cost of the energy-adjusted NND is about 16-17% higher than the ADD, both in the reported and the behaviourally adjusted versions of the two diets, with the smallest difference in the behaviourally adjusted version.

Table 4 shows the 10 most significant food budget items (out of a total of 83 food and beverage categories) in the reported and behaviour-adjusted versions of the two diets, in terms of cost. In both versions of the ADD, meats play important roles, with meats constituting 4 of the 10 most significant itmes in the food and beverage budget, while vegetables and seafood products being completely absent. In contrast, vegetables (especially root and leaf vegetables and herbs) and fish products are among the most significant items in the NND budget (in both versions).

**Table 4:** Top-10 cost items in the food and beverage budget in the reported and behaviour-adjusted ADD and NND

**ADD-i**	**ADD-m**	**NND-i**	**NND-m**
Beef	Beef	Root vegetables	Bread
Sugar products	Sugar products	Bread	Pomes
Soured milk products	Soured milk products	Leaf vegetables	Salt water fish
Bread	Bread	Pomes	Leaf vegetables
Lunch meats	Lunch meats	Salt water fish	Root vegetables
Tropical fruit	Tropical fruit	Herbs	Herbs
Pork	Pork	Processed fish	Processed fish
Pomes	Pomes	Lemonade etc.	Lemonade etc.
Hard cheese	Fresh milk products	Lunch meats	Fresh milk products
Chicken	Chicken	Fresh milk	Berries

Note: Out of 83 commodity groups.

**Figure 1:**
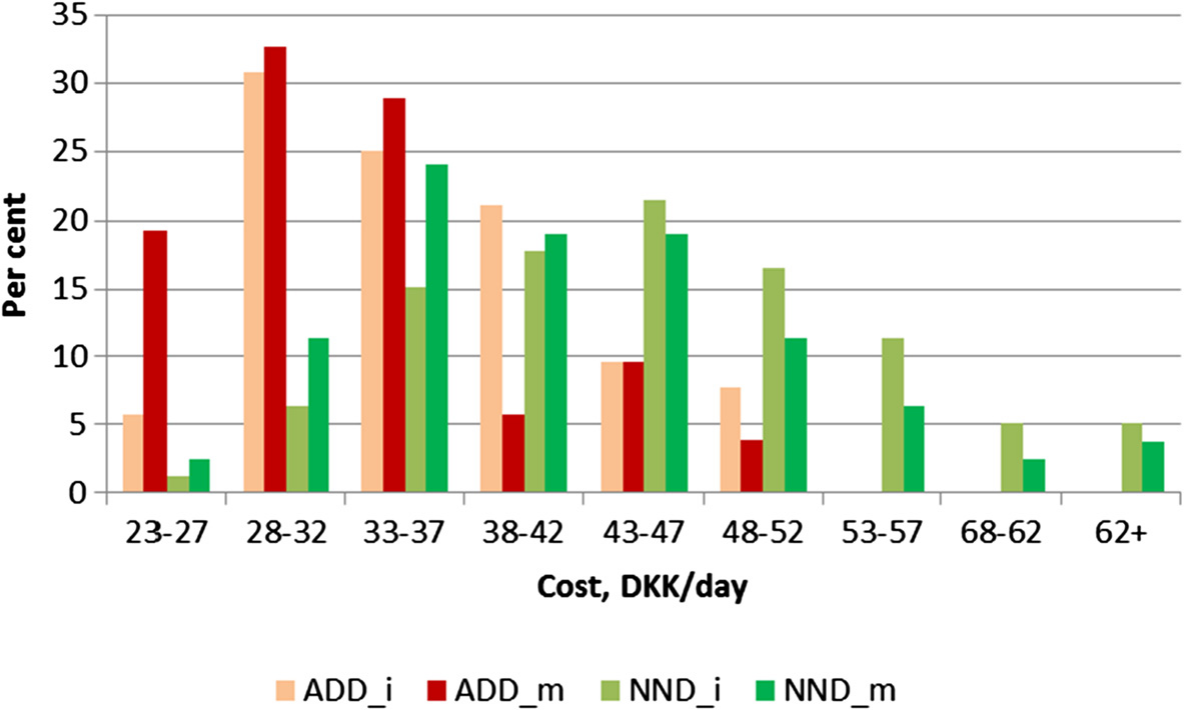
Distribution of daily dietary costs in the four diet scenarios.

The data in Table 3 represent average daily costs of the respective diets, as modeled on the basis of the intervention. It is however also interesting to look more into the variation of these costs among consumers (i.e. participants in the intervention). This variation is displayed in Figure 1, which shows the distribution of participants according to daily food expenditure for the four diet scenarios. For the two ADD-scenarios, the bulk of the participants (60-80%) seem to be relatively homogenous in terms of food expenditure and to exercise a food pattern that would cost them in the area of 25–35 DKK per day – and especially so in the adjusted ADD scenario. The distribution of the adjusted ADD-scenario is positioned to the left of the unadjusted ADD-scenario, which was also expected, because the adjustment reflects consumers’ seeking for a lower food cost within the framework of the considered diet.

Interestingly, the dietary costs in the New Nordic Diet are much more heterogeneous among consumers than in the ADD-scenarios, and with a significantly higher share of the participants exhibiting relatively high daily food costs. A majority of the participants (50-60%) have a food pattern that represents a daily cost in the range of 35–45 DKK, and there is a relatively large share of the participants using foods for more than 50 DKK/day in the NND-I, whereas a relatively small share of the participants use foods for less than 35 DKK/day. Also in the case of the New Nordic Diet, the distribution for the adjusted diet is located to the left of the unadjusted diet.

## Discussion

By combining quantity data from the intervention with estimated market price data from household panel purchase data, it has been possible to calculate an estimate of the daily food cost for the consumers. The results suggest that 100 per cent adherence to the New Nordic Diet is on average 24–25 per cent more costly than the Average Danish Diet (and 16–17 per cent when adjusting for energy content), but also that the variation in daily food expenditure is larger in the New Nordic Diet than in the Average Danish Diet, and that a relatively large share of the participants randomized to the NND treatment consumed foods for considerably more than the ADD mean of about 33–36 DKK/day. An adherence less than 100 per cent to the NND might reduce the additional costs proportionately, however depending on the composition of NND- and ADD elements in such a ‘mixed’ diet.

These findings imply that it might be possible to feed the population with the New Nordic Diet at a cost 24–25 per cent higher than the normal food budget, corresponding to an annual food budget increase of about 7–8000 DKK for an average Danish household. These costs should be compared with the likely health benefits, which may be expected to be derived from a diet in line with nutritional recommendations [23]. Such health benefits might also compensate the individuals for (some of) the extra costs of the New Nordic Diet. On the other hand, the NND tends to be more reliant on basic cooking efforts than the ADD, suggesting that the NND may lack some of the convenience attributes of the ADD, and that this may constitute an implicit cost – and hence a barrier - for some consumers. As large shares of the Danish population are currently choosing a diet that deviates from the New Nordic Diet, many consumers do not seem to perceive these benefits as sufficient to compensate for the higher cost. There may thus be an important challenge to enhance consumers’ perception of the New Nordic Diet, through information, increased availability, facilitation, etc.

In addition to health benefits, a study by Saxe et al. suggests that the New Nordic Diet might also have a lower climate impact than the Average Danish Diet [28]. However, in contrast to health benefits of improved nutrition, the individuals’ incentives to contribute to such climatic benefits are expected to be lower, due to the public-good nature of climate effects. Such incentives could be strengthened by imposing regulations on high-carbon foods (such as meat), for example in the form of carbon taxes on foods.

It should be kept in mind that the food choices made by the participants in the intervention to a significant extent have been programmed by the prescribed diets, and furthermore may have been biased by the fact that they got the foods for free and hence might have a stronger incentive to choose “expensive” ingredients than would have been the case, if they had to pay the full price. It should also be kept in mind that the participants in the intervention were overweight, which may have an influence on their general food intake. On the one hand, they may have a higher energy requirement (which would suggest an above-average food intake), but on the other hand they might also have a desire to lose weight as part of their participation in the intervention (which might suggest a below-average intake). Figures from Statistics Denmark’s household consumption surveys suggest that the daily expenditure per “adult-equivalent” (where children count as 0.6 adult-equivalents) was about 45 DKK in the period 2009–2011, but that the composition of the food budget is quite similar to the ADD. Thus, the comparison with official statistical data suggests that the average food expenditure in the intervention may tend to be under-estimated in both the NND and the ADD. This might be due to the fact that the intervention data have been “cleaned”, whereas the above-mentioned official statistical data leading to an average daily expenditure of 45 DKK per adult represent total purchases from retail stores, including foods that are wasted in the households. But if the extent of under-estimation is similar in the two diets, the relative difference between the two may still be estimated adequately.

Reduction of food waste is an element in the sustainability dimension of the NND, where improved utilization of left-overs is integrated in the recipes developed for the intervention. The NND is not a prerequisite for lower food waste (compared to ADD), but it could be imagined that the higher extent of own-preparation in the NND would enable better utilization of the ingredients - and perhaps also increase the consumers' motivation to avoid food waste.

It should also be noted that seasonal variation in ingredient availability and food prices may also have affected the results. The intervention period commenced in the time span between October 2010 and January 2011, and (for individuals completing the interventions) ended in the time interval between April and July 2011. Hence, especially the NND tends to be dominated by winter and spring dishes. It might be presumed that the cost differential would be different in the summer and early autumn season, which is also harvesting season for many types of fruits and vegetables.

The finding that the energy-adjusted NND is approximately 16–17 per cent more costly than the corresponding ADD is fairly consistent with findings from previous studies in the national and international literature. Stender et al. [22] found that a reduction of dietary fat from 35 E% to 25 E% might increase food costs by 10-20% for Danish children, Rydén & Hagfors [5] also found that a healthy diet is about 15% more costly than a less healthy diet in Sweden, and Schröder et al. [4] found that the average daily cost of adhering to the Mediterranean diet was 1.2€ and for adhering to a satisfactory Healthy eating index was 1.4€ (each corresponding to 17-18% of the non-adherence daily food cost) for Spanish adults.

Drewnowski et al. [16] found that a low-energy-density diet was about 10 per cent more expensive than a high-energy density diet among French adults, and Townsend et al. [19] found a cost difference of about 20 per cent between a low- and a high-energy density diet in California. However, Ottelin et al. [20] did not find significant differences in dietary cost between the intervention and the control group, and neither did Raynor et al. [21] find significant differences in the cost per MJ in a weight-loss diet, compared with a “normal” diet.

## Conclusion

The present study finds that the New Nordic Diet is on average 24–25 per cent more expensive than an Average Danish Diet (or 16–17 per cent when adjusting for energy content) at the current market prices in Denmark, which is similar to results of previous studies of the costs of a healthy diet, compared with a less healthy diet. Furthermore, we find that the distribution of food cost is much more heterogeneous among consumers within the NND than within the ADD, suggesting some possibility for adhering to the NND at a lower additional cost than the 24–25 per cent. To some extent, these extra costs may be compensated by higher gastronomic quality, as well as by improved health prospects.

### Ethical approval

Ethical approval not required.

## Abbreviations

OPUS: Acronym for the project ‘Optimal well-being, development and health for Danish children through a healthy New Nordic Diet’; NND: New nordic diet; NND-i: New nordic diet, consumed quantities as measured in intervention; NND-m: New nordic diet, consumed quantities adjusted to market price relations; ADD: Average danish diet; ADD-i: Average danish diet, consumed quantities as measured in intervention; ADD-m: Average danish diet, consumed quantities adjusted to market price relations.

## Competing interests

The authors declare that they have no competing interests.

## Authors’ contributions

JDJ planned and conducted the analysis and drafted the manuscript. SP managed the intervention and collected data from the intervention. Both authors read and approved the final manuscript.

## Pre-publication history

The pre-publication history for this paper can be accessed here:

http://www.biomedcentral.com/1471-2458/13/1114/prepub
